# Eu-doped carbon quantum dot as a selective probe for visualizing and monitoring sulfite in biological systems

**DOI:** 10.3389/fbioe.2023.1292136

**Published:** 2023-12-08

**Authors:** Bo Kan, Li Li, Jiaoyu Hou, Shuyan Liu, Zhenwei Tian, Qianchuang Sun

**Affiliations:** ^1^ Department of Clinical Laboratory, The Second Hospital of Jilin University, Changchun, Jilin, China; ^2^ College of Chemistry, Jilin University, Changchun, Jilin, China; ^3^ Department of Geriatrics, The First Hospital of Jilin University, Changchun, Jilin, China; ^4^ Department of Ophthalmology, The Second Hospital of Jilin University, Changchun, Jilin, China; ^5^ Department of Emergency and Critical Care, The Second Hospital of Jilin University, Changchun, China; ^6^ Department of Anesthesiology, The Second Hospital of Jilin University, Changchun, Jilin, China

**Keywords:** fluorescent probe, selectivity, cellular imaging, sulfite detection, biocompatibility

## Abstract

The detection of SO_3_^2−^ in complex environments and its visualization at the cellular level are critical for understanding its role in biological processes. In this study, we developed an Eu-doped long-wavelength fluorescent carbon quantum dot (CD2) and investigated the detection mechanism, interference effects and cellular imaging applications of the fluorescent probe CD2. The results show that the addition of SO_3_^2−^ induces an electronic rearrangement that restores CD2 to its original structure, leading to a rapid increase in fluorescence intensity. Selectivity experiments showed that CD2 has excellent selectivity to SO_3_^2−^, with minimal interference from common anions. In addition, CD2 shows good biocompatibility for cellular imaging applications, as evidenced by the high cell viability observed in HeLa cells. Using confocal microscopy, we detected a significant enhancement of red fluorescence in HeLa cells after addition of exogenous SO_3_^2−^, demonstrating the potential of CD2 as a probe for monitoring cellular SO_3_^2−^ levels. These findings highlight the promise of CD2 as a selective SO_3_^2−^ detection probe in complex environments and its utility in cellular imaging studies. Further studies are necessary to fully exploit the potential of CD2 in various biological and biomedical applications.

## 1 Introduction

The detection of intracellular sulfite ions (SO_3_^2−^) is of great significance in the field of biomedical engineering (Tian et al., 2013; Wang et al., 2019; Li Q. et al., 2023). These ions play a critical role in various cellular processes and provide valuable insights into cell function and health. Accurate measurement of sulfite ions is crucial for disease diagnosis, drug development, and understanding the impact of environmental factors on cellular function (Maiti, 2022; Săcărescu et al., 2023).

Within cells, sulfite ions are generated through the oxidation of sulfur-containing amino acids, including cysteine and methionine. These ions are involved in critical intracellular processes such as protein folding, enzyme regulation, and antioxidant defense mechanisms (Buerman et al., 2021). By modulating cellular signaling pathways, sulfite ions influence gene expression and cellular responses to a wide range of stimuli.

Maintaining an appropriate balance of intracellular sulfite ions is essential for optimal cell health and functionality (Kimura et al., 2019). Imbalances in sulfite ion levels have been associated with the occurrence and progression of several diseases, including cardiovascular disorders, neurodegenerative diseases, and cancer (Zhang et al., 2004). Elevated sulfite concentrations can lead to oxidative stress and cell damage, as excess sulfite can react with reactive oxygen species (ROS) to form toxic compounds (Mitsuhashi et al., 2005). Conversely, abnormally low sulfite ion levels can disrupt cellular processes dependent on sulfite signaling, resulting in impaired antioxidant defenses and compromised cell function (Vincent et al., 2004).

Accurate measurement of intracellular sulfite ion concentrations is instrumental in biomedical research and applications. Sulfite ion detection enables the assessment of sulfite-related diseases, aids in diagnosis, and guides the development of targeted treatment approaches. Additionally, it provides a means to evaluate the impact of potential therapeutic compounds on sulfite-mediated signaling pathways, thereby contributing to advancements in drug development (Danışman et al., 2022; Guo et al., 2023).

Various techniques have been developed for the quantitative detection of intracellular sulfite ion levels (Zhang et al., 2016; Wang P. et al., 2021). Prominent examples include fluorescence-based detection methods (Fu et al., 2022), mass spectrometry analysis (Robbins et al., 2015), and electrochemical biosensors (Badihi-Mossberg et al., 2007). Fluorescent probes allow for real-time visualization and monitoring of sulfite ions within live cells, while mass spectrometry offers high sensitivity and accurate measurements. Electrochemical biosensors provide a simple and rapid detection method suitable for point-of-care applications.

**SCHEME 1:**
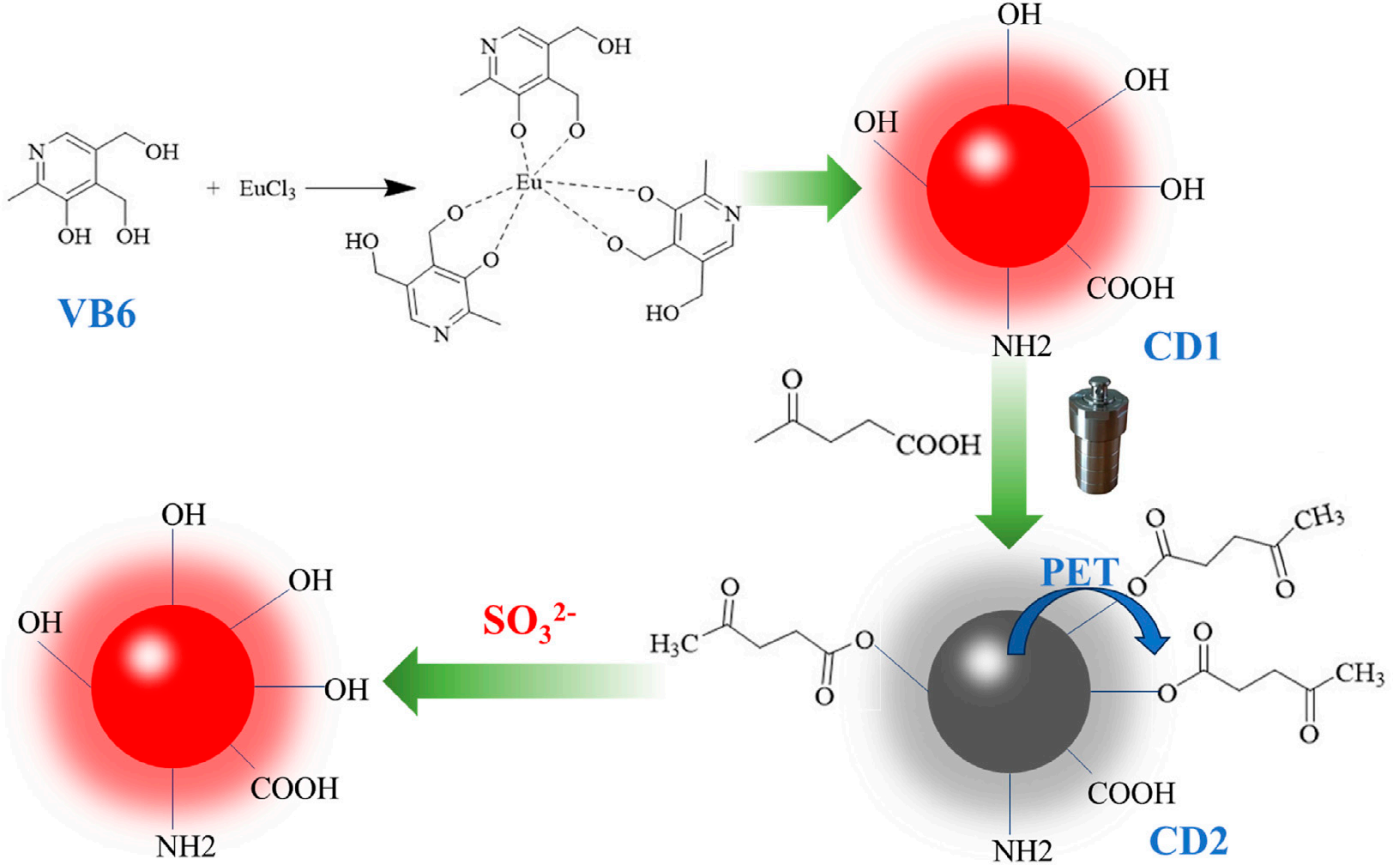
The demonstration diagram of the preparation of carbon dots and the process of SO_3_^2−^ detection.

In this study, we propose a novel approach for sulfite ion detection by synthesizing long-wavelength emission fluorescence carbon quantum dots (CD1). These CD1 dots, derived from pyridoxine (vitamin B6) as the carbon source and doped with Eu^3+^, possess exceptional properties such as low toxicity, high biocompatibility, and intense fluorescence (Li et al., 2021; Wareing et al., 2021; Ðorđević et al., 2022). To achieve sulfite ion detection, the CD1 dots are subjected to surface modification with pyruvic acid, which quenches their fluorescence through the photoinduced electron transfer (PET) effect (Sun et al., 2019; Allen et al., 2022), rendering them non-fluorescent (hereafter referred to as CD2). Upon reaction with sulfite ions, the removal of pyruvic acid from the surface of CD2 leads to a considerable fluorescence enhancement (Deng et al., 2019; Li W.-B. et al., 2023), enabling highly sensitive detection of sulfite ions (Scheme 1).

Accurate measurement of sulfite ions is vitally important for disease diagnosis, drug development, and understanding the influence of environmental factors on cellular function. The proposed synthesis of long-wavelength emission fluorescence carbon quantum dots, coupled with surface modification, presents a promising approach for achieving sensitive and reliable detection of sulfite ions.

## 2 Materials and methods

### 2.1 Materials and instrumentation

Pyridoxine, EuCl_3_, Na_2_SO_3_ and other compounds were unpurified using commercially available standard chemicals. Deionized water used in the experiments was prepared in the laboratory.

Transmission electron microscopy (TEM) and high-resolution TEM (HRTEM) images of the CDs were recorded using a JEOL-2100F. Fourier transform infrared spectra (FTIR) were obtained using a Nicolet IS-10 FTIR spectrophotometer (Thermo Fisher Scientific). Fourier transform infrared spectra (FTIR) were obtained using a Nicolet IS-10 FTIR spectrophotometer (Thermo Fisher). X-ray photoelectron spectroscopy (XPS) was performed using an X-ray photoelectron spectrometer (AXIS ULTRA DLD). UV-vis absorption spectra were tested on an Agilent Cary 300 Scan. The zeta potential and hydrodynamic size of the samples were determined using a Zetasizer Nano ZS-90 analyzer (Malvern Instruments). Absolute QY and lifetimes were determined using a FLS1000 instrument (Edinburgh Instruments). Fluorescence spectra were obtained using an F97 Pro fluorescence spectrophotometer. Cells were imaged using a Leica TCS-SP8 confocal microscope.

### 2.2 Preparation of the CD1 and CD2

0.5 g of pyridoxine and 0.1 g of EuCl3 were dissolved in 40 mL of deionized water and sonicated for 10 min, then the solution was transferred to a 100 mL Teflon-lined stainless-steel autoclave. CD1 solution was obtained after cooling. To the CD1 solution 0.3 g of pyruvic acid was added, stirred well and returned to the reactor for secondary heating for 4 h (130°C). After cooling to room temperature, the reaction product was eluted with a mixture of dichloromethane and methanol (10:1) to give a bright red liquid CD2, which was dried by rotary evaporator. The dried solid was stored at 4°C in a dark place for future characterization and use.

### 2.3 Detection of SO_3_^2-^

The fluorescence spectra of the samples were recorded after adding different concentrations of SO_3_^2−^ (1–25 μM) to the CD2 solution at 20 μg/mL.

### 2.4 Cytotoxicity assays and cell targeting

The cytotoxicity of CD2 was tested using WST-1. HeLa cells were cultured in 96-well plates at 10^4^ cells per well. The culture medium consisted of 10% FBS, 100 U/mL penicillin-streptomycin solution and 90% RPMI 1640 medium. After 24 h of incubation, the medium was removed, fresh complete medium containing different concentrations of CD2 (dissolved in DMSO, 5 mg/mL) was added, and the cells were incubated for another 24 h. Finally, the medium was removed, and the cells were rinsed three times with PBS, and the viability of the cells was assessed by the WST-1 assay. Absorption was recorded for all plates using a microplate reader with a wavelength of 450 nm.

To assess the ability of CD2 to detect intracellular SO_3_^2−^, HeLa cells were seeded into 35-mm glass-bottomed Petri dishes and cultured at 37°C in a humidified environment with 5% CO_2_. After 24 h of incubation, the initial medium was removed, and then 20 μg/mL of CD2 was added with 2 mL of fresh medium for another 6 h. An additional 25 μM of SO_3_^2−^ was added to the control experiments. Then, after rinsing the cells three times with PBS, the fluorescence images of the samples were observed using a laser fluorescence confocal microscope.

## 3 Results and discussion

### 3.1 Preparation and characterization of the CDs

**FIGURE 1:**
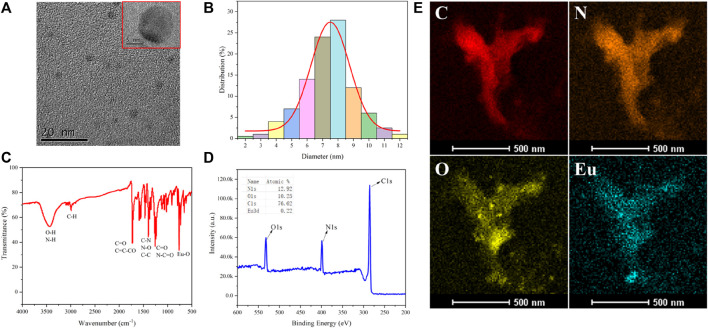
**(A)** TEM picture of CD1; **(B)** DLS particle size distribution; **(C)** IR spectra; **(D)** XPS analysis; **(E)** EDS elemental analysis.

CD1 was synthesized through a hydrothermal reaction using VB6 as the carbon source and Eu^3+^ as the dopant. CD1 possesses hydroxyl groups on its surface, which can be easily reacted with pyruvic acid through a two-step hydrothermal process. By utilizing the photoinduced electron transfer (PET) effect, the fluorescence of CD1 can be quenched. TEM analysis (Figure 1A) revealed that CD1 exhibited a regular spherical structure with a uniform size and single dispersion in water. The individual particle diameter was determined to be approximately 8 nm, which was further confirmed by DLS measurements (Figure 1B).

The infrared spectrum of CD1 (Figure 1C) exhibited several characteristic peaks. The peak at around 750 cm^−1^ originated from the stretching vibration of Eu-O bonds, indicating the successful doping of Eu^3+^ into the CD1 structure. The peak at 1,240 cm^−1^ represented the stretching vibration of C=O and N-C=O bonds, while the peak at 1,396 cm^−1^ was attributed to the stretching vibration of C-N bonds and the bending vibration of N-O bonds. The peak at 1716 cm^−1^ indicated the presence of the C=C-CO bending vibration, suggesting the existence of conjugated olefin or aromatic ketone structures in CD1. The peak at 2,992 cm^−1^ represented the stretching vibration of C-H bonds and alkynyl groups. The peak at 3,423 cm^−1^ corresponded to the stretching vibration of O-H and N-H bonds, indicating the presence of hydroxyl and amino groups in CD1. X-ray photoelectron spectroscopy (XPS) analysis (Figure 1D) confirmed that CD1 primarily consisted of carbon (C), nitrogen (N), and oxygen (O), with a small amount of Eu (atomic percentage of 0.22%). Furthermore, energy-dispersive X-ray spectroscopy (EDS) elemental analysis (Figure 1E) provided additional confirmation that CD1 was composed of C, N, O, and Eu elements.

Overall, the results demonstrate the successful synthesis of CD1 with a well-defined structure and composition. The incorporation of Eu^3+^ as a dopant and the surface modification with pyruvic acid provide CD1 with favorable properties for sulfite ion detection.

### 3.2 Optical properties of carbon dots

**FIGURE 2:**
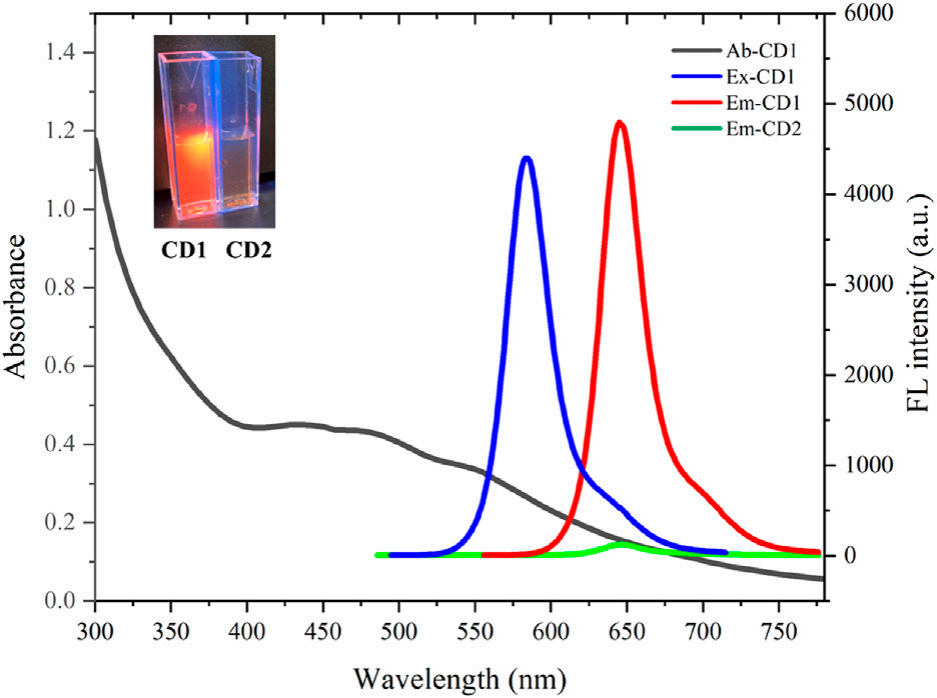
Absorption (black line), excitation (blue line) and emission (red line) spectra of CD1; emission (green line) of CD2.

The optical properties of the carbon dots are depicted in Figure 2. CD1 shows absorption throughout the visible light range, with a shoulder peak around 470 nm. Its emission peak is located at 650 nm, with an optimal excitation peak at 580 nm. In contrast, CD2 exhibits nearly no fluorescence, with emission intensity even less than 1/50th of CD1’s. Thus, CD1 displays bright red fluorescence under excitation, while CD2 exhibits minimal fluorescence (shown in the top left corner of the image). This indicates that the reaction of pyruvic acid with CD1 through the secondary hydrothermal process leads to the quenching of its fluorescence.

The significant contrast in fluorescence between CD1 and CD2 provides the basis for the detection of sulfite ions. The presence of sulfite ions can cause the removal of pyruvic acid from the surface of CD2, restoring its fluorescence. This fluorescence enhancement can be utilized for sensitive and selective detection of sulfite ions. The changes in the optical properties of the carbon dots upon interaction with sulfite ions will allow for the quantification of sulfite ion concentrations in various biological and environmental samples.

### 3.3 Detection of SO_3_^2-^ in solution

**FIGURE 3:**
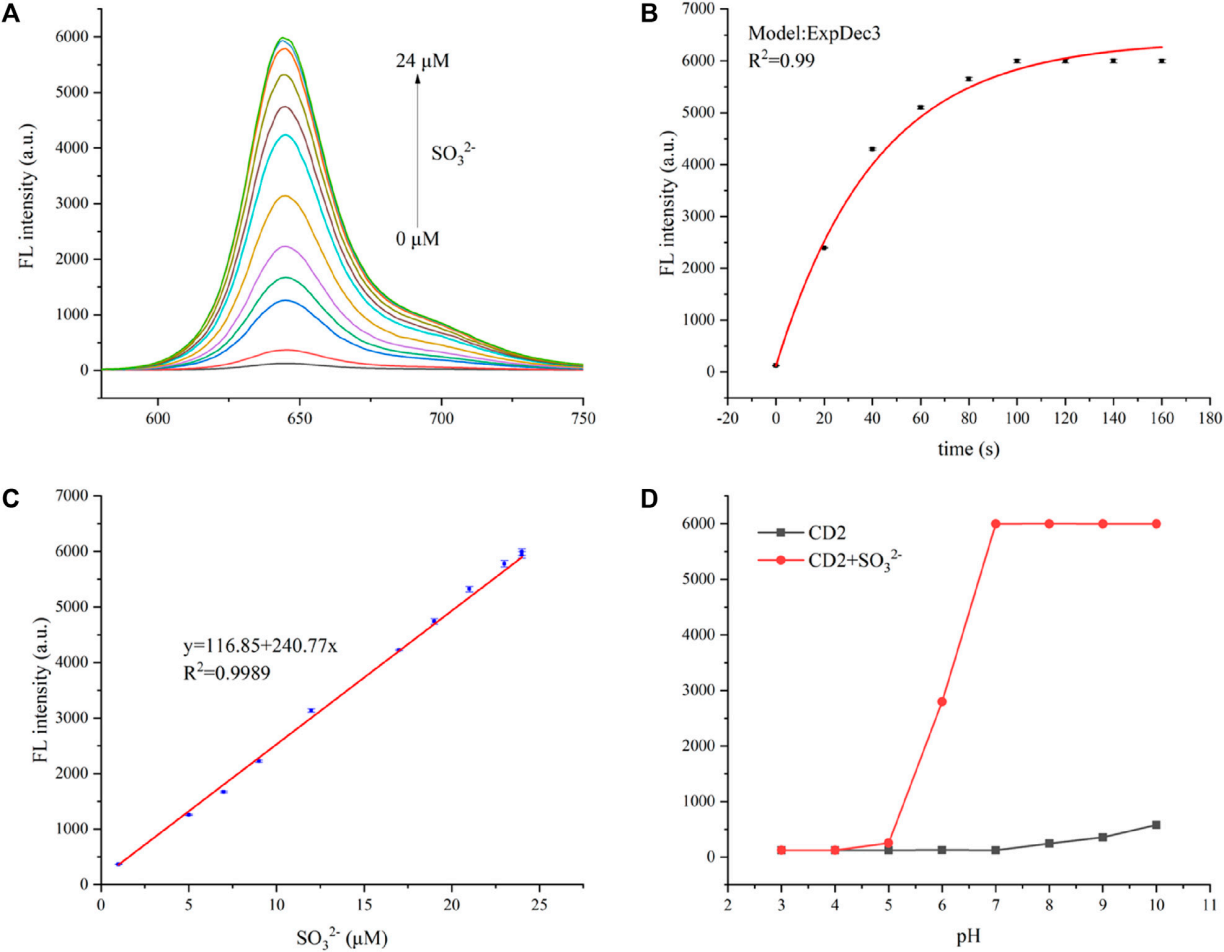
**(A)** Fluorescent spectra of the CD2 solution with the addition of different concentrations of SO_3_^2−^ at an excitation wavelength of 580 nm. **(B)** Time-dependent fluorescence intensity spectra of CD2 (20 μg/mL) at 650 nm in the presence of SO_3_^2−^ (25 µM). **(C)** The linear relationship between (F0/F—1) and the concentration of SO_3_^2−^ over the range of 1–25 µM. **(D)** The fluorescence intensity of CD2 (20 μg/mL) at 650 nm in the absence and presence of SO_3_^2−^ (25 µM) in various pH conditions (3.0–10.0). Incubation temperature: 37°C.

The fluorescence-quenched CD2 has been utilized for highly sensitive detection of SO_3_^2−^. A certain concentration of CD2 aqueous solution (20 μg/mL) was prepared, and different concentrations of SO_3_^2−^ (ranging from 1–25 µM) were added. As the concentration of SO_3_^2−^- increased, the fluorescence intensity of the solution rapidly intensified. Even with the addition of 5 µM SO_3_^2−^, the fluorescence intensity increased by more than six times. When 25 µM SO_3_^2−^ was added, the intensity increase slowed down, eventually reaching over 30 times the initial intensity (Figure 3A).

The fluorescence of the CD2 solution enhanced almost instantaneously upon the addition of SO_3_^2−^, reaching stability within 2 min (Figure 3B). This indicates the high sensitivity of CD2 to SO_3_^2−^ and its potential as a rapid detection probe. Figure 3C displays the relationship between SO_3_^2−^ concentration and the fluorescence intensity of the CD2 solution. It shows good linearity within the range of 1–20 µM (*R* ^2^ = 0.998), following equation *F/F*_*0*_—1 = 0.429 + 1.968x, where *F* represents the fluorescence intensity of the CD2 solution after the addition of SO_3_^2−^, and *F*_*0*_ represents the initial fluorescence intensity of the CD2 solution.

To determine the limit of detection (LOD), the equation LOD = 3σ/S was used, in which σ is the standard deviation of the fluorescence intensity of the CD2 solution without the addition of SO_3_^2−^, and S is the slope of the fitting line. Based on this calculation, the LOD was estimated to be approximately 0.15 µM, which is lower than both electrochemical and colorimetric methods, and at the same level as other fluorescent probes (Table 1). And the limit of quantification (LOQ) was calculated as 0.5 µM.

**TABLE 1:** Comparison with existing SO_3_^2−^ detection methods.

Materials	Method	Linear range	LOD (µM)	References
Nanostructured copper-salen film	electrochemical sensor	4–69 µM	1.2	Dadamos and Teixeira (2009)
Ag_2_O nanoparticles	colorimetric detection	100–500 µM	10	Lu et al. (2015)
rhodamine-derivated probe	fluorescent	0–80 µM	0.6	Wang et al. (2021a)
ratiometric fluorescent probe	fluorescent	0–100 µM	0.57	Venkatachalam et al. (2020)
Carbon dots	fluorescent	0–25 µM	0.15	This work

Meanwhile, the effect of pH on the detection process was considered. The probe CD2 itself is almost non-fluorescent at different pH values, and the detection of sulfites remains stable in the pH range of 7.0–10.0 (Figure 3D). The pH values of human sulfite metabolism sites are mainly between 7.0 and 8.5 (e.g., brain 7.1, heart 7.0–7.4, bile 7.8, liver 7.2, blood 7.3–7.45, pancreatic secretion 8.0–8.3, and bone 7.4) (Hashim et al., 2011; Yue et al., 2017), and therefore, CD2 is almost sufficient for the detection.

These results suggest that the quenching and recovery of fluorescence in CD2 can be effectively applied for the sensitive and selective detection of sulfite ions in solution. The high sensitivity, rapid response, and low limit of detection make CD2 a promising candidate for sulfite ion detection in various applications.

### 3.4 Mechanisms for the detection of SO_3_^2-^

**FIGURE 4:**
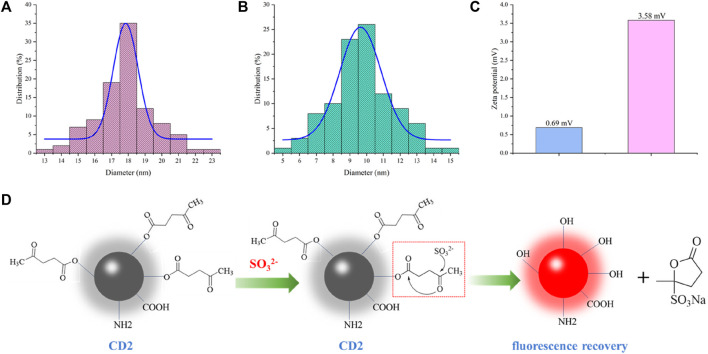
**(A)** Particle size of CD2 before addition of SO_3_^2−^; **(B)** Particle size of CD2 after addition of SO_3_^2−^; **(C)** Zeta potential of CD2 before and after addition of SO_3_^2−^; **(D)** Predicted detection mechanism.

To gain insight into the detection mechanism of SO_3_^2−^, we conducted tests on the particle size (Figures 4A, B) and surface potential (Figure 4C) of the solution before and after the reaction. Upon the addition of SO_3_^2−^, the particle size of CD2 decreased from approximately 18 nm to around 10 nm, which is close to the average particle size of CD1. Additionally, the surface potential increased from +0.69 mV to +3.58 mV. These observations provide valuable information for proposing a possible detection mechanism for SO_3_^2−^ (Figure 4D).

In the two-step hydrothermal method, pyruvic acid is chemically modified onto the surface of CD1 through an ester linkage, resulting in the formation of CD2. The fluorescence of CD2 is quenched due to the photoinduced electron transfer (PET) effect. Upon the addition of SO_3_^2−^ to the CD2 solution, the oxygen atom in SO_3_^2−^ interacts with the aldehyde group of pyruvic acid. This interaction leads to electron rearrangement and the formation of a transition state involving the oxygen atom on the carbonyl carbon of the ester moiety. Finally, the transition state decomposes, breaking the ester bond and generating a sulfonate-substituted furanone derivative. As a result, CD2 is restored to the structure of CD1, and the fluorescence intensity rapidly increases.

Further investigations are necessary to fully understand the detailed mechanism of SO_3_^2−^ detection using CD2. Nevertheless, these preliminary findings provide important insights and a plausible explanation for the observed fluorescence enhancement upon the addition of SO_3_^2−^ in the CD2 solution.

### 3.5 Interference experiments

**FIGURE 5:**
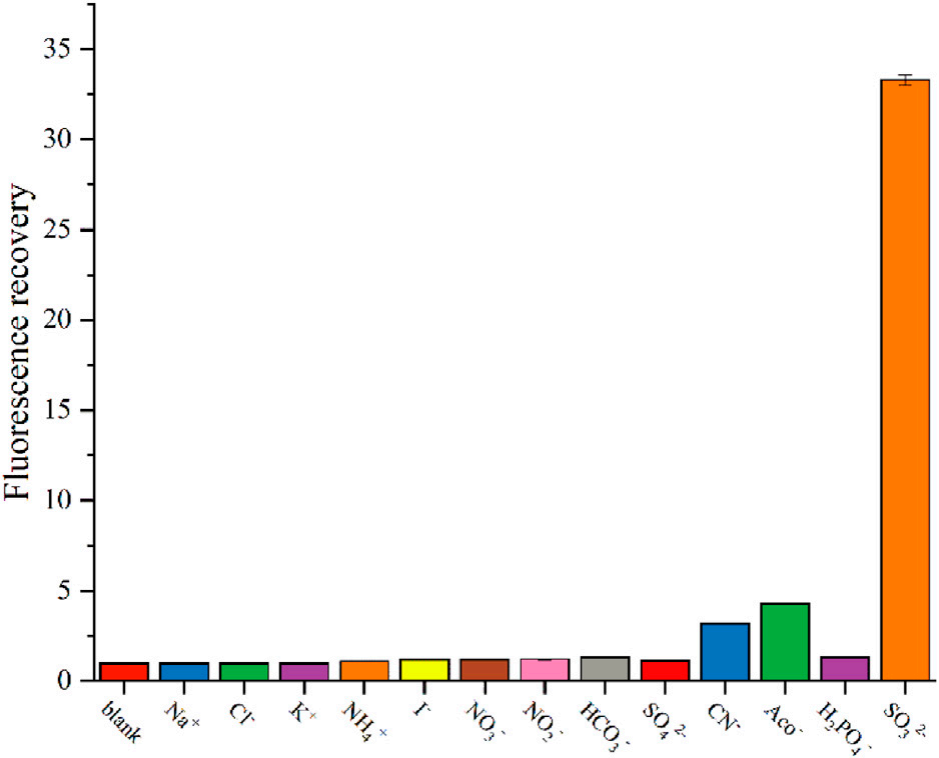
Change in fluorescence intensity of CD2 solution after addition of interfering substances.

To assess the interference of other ions in the detection process of SO_3_^2−^, we conducted experiments to test the effects of commonly encountered anions, such as Cl^−^, I^−^, NO^3−^, HCO^3−^, SO_4_^2−^, AcO^−^, CN^−^, and H_2_PO_4_^−^, on the fluorescence of the CD2 solution. Figure 5 illustrates the results obtained.

Upon adding 25 μM of Na^+^, K^+^, NH_4_^+^, Cl^−^, I^−^, NO^3−^, HCO^3−^, SO_4_^2−^, and H_2_PO_4_^−^ individually, the fluorescence intensity of the CD2 solution remained almost unchanged. Most of common cations and anions do not interfere with the detection, consistent with other research (Wang K. et al., 2021). Only CN^−^ and AcO^−^ caused a slight increase in fluorescence intensity, approximately three times higher. However, when comparing this increase to the 30-fold amplification observed with SO_3_^2−^, it is negligible.

These findings indicate that CD2 exhibits exceptional selectivity for SO_3_^2−^. The lack of interference from commonly encountered anions suggests that the detection of SO_3_^2−^ in complex environments holds promising potential for various applications.

### 3.6 Toxicity assays and cellular imaging applications of CD2

To assess the feasibility of using CD2 for cell imaging, a cell viability assay was conducted using the WST-1 reagent. The results demonstrated that the cell viability of HeLa cells remained above 90% when different concentrations of CD2 (ranging from 0 to 100 μg/mL) were added. This indicates that CD2 exhibits good biocompatibility for cell imaging purposes.

**FIGURE 6:**
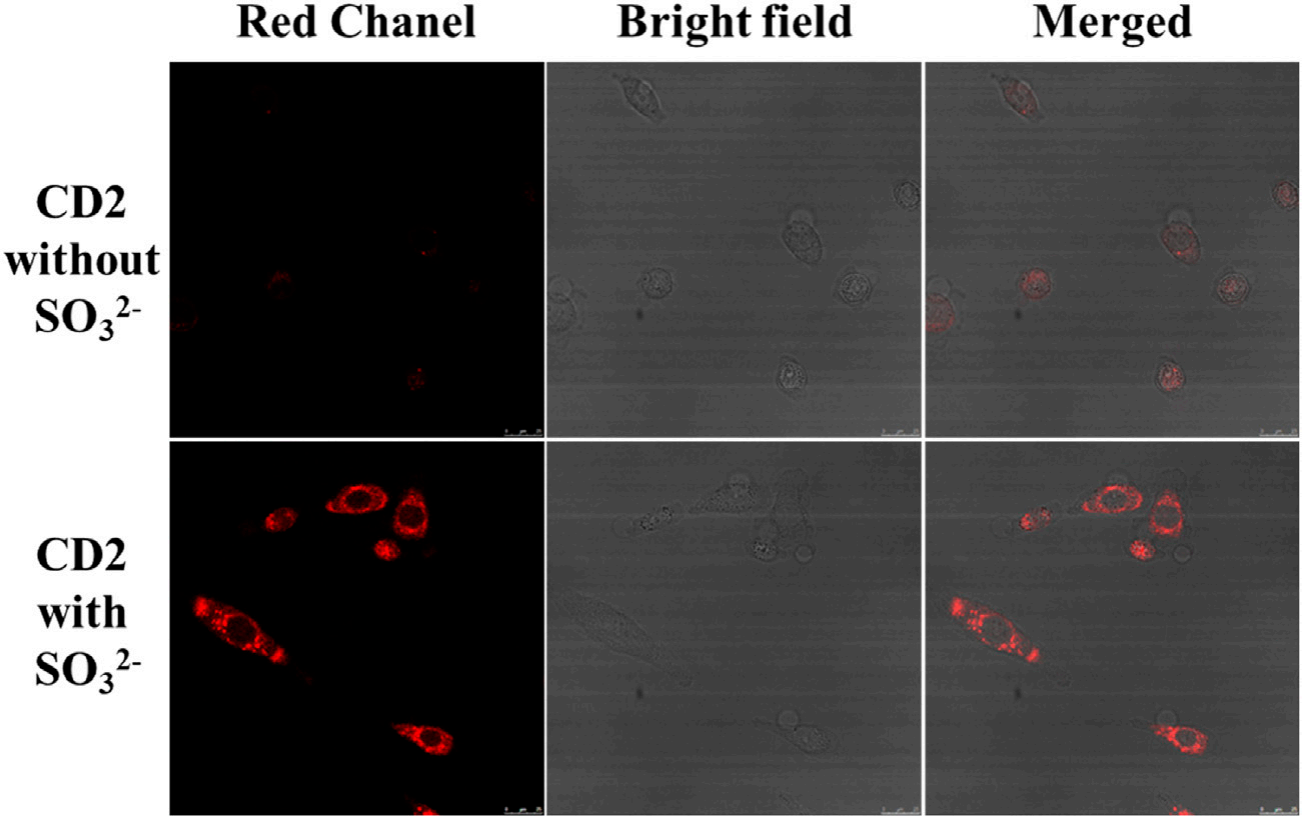
Changes in fluorescence intensity of HeLa cells before and after addition of SO_3_^2−^ (scale bar: 25 μm).

Subsequently, the potential application of CD2 in cell imaging was further explored using a Leica SP8 confocal microscope. The fluorescence changes of CD2 were studied at the cellular level by introducing exogenous SO_3_^2−^ into HeLa cells. Figure 6 illustrates the results obtained. As shown, a significant enhancement of red fluorescence was observed in HeLa cells following the addition of 20 μM of SO_3_^2−^. This observation suggests that CD2 can effectively detect the level of SO_3_^2−^ in HeLa cells and has the potential to serve as a probe for cellular imaging applications.

These findings demonstrate the potential of CD2 for cellular imaging and highlight its ability to detect and monitor the levels of SO_3_^2−^ in living cells.

## 4 Conclusion

In conclusion, our study investigated the detection mechanism, interference effects, and potential applications of CD2 in the detection of SO_3_^2−^. The results indicated that CD2 exhibits a promising detection mechanism, where the addition of SO_3_^2−^ leads to electron rearrangement and the restoration of CD2 to its original structure, resulting in a rapid increase in fluorescence intensity. Furthermore, CD2 demonstrated excellent selectivity for SO_3_^2−^, as evidenced by minimal interference from commonly encountered anions.

To assess the biocompatibility of CD2 for cellular imaging applications, a cell viability assay exhibited high cell viability when different concentrations of CD2 were added to HeLa cells. This indicates that CD2 has good biocompatibility for cell imaging. Moreover, cellular imaging experiments using CD2 revealed its ability to detect and monitor the levels of SO_3_^2−^ in living cells, as manifested by a significant enhancement of red fluorescence upon the addition of exogenous SO_3_^2−^ to HeLa cells.

Overall, these findings suggest that CD2 holds promise as a probe for the detection of SO_3_^2−^ in complex environments and its application in cellular imaging. Further studies are warranted to explore the full potential and specific mechanisms of CD2 in various applications.

## Data Availability

The original contributions presented in the study are included in the article/supplementary material, further inquiries can be directed to the corresponding author.
